# Prenatal Intuitive Coparenting Behaviors

**DOI:** 10.3389/fpsyg.2016.01662

**Published:** 2016-10-27

**Authors:** Joëlle Darwiche, Elisabeth Fivaz-Depeursinge, Antoinette Corboz-Warnery

**Affiliations:** ^1^Family and Development Research Center (FADO), Institute of Psychology, University of LausanneLausanne, Switzerland; ^2^Research Unit of the Centre for Family Study, University Institute of Psychotherapy, University of LausanneLausanne, Switzerland

**Keywords:** Prenatal Lausanne Trilogue Play, coparenting, observation, parenting, intuitive parenting behaviors

## Abstract

Micro-analytic research on intuitive parenting behaviors has shed light on the temporal dynamics of parent and child interactions. Observations have shown that parents possess remarkable implicit communicative abilities allowing them to adapt to the clues infants give and therefore stimulate the development of many of the infants’ abilities, such as communication skills. This work focused on observing intuitive parenting behaviors that were synchronized and coordinated between the parents. We call them “prenatal intuitive coparenting behaviors” and used an observation task – the Prenatal Lausanne Trilogue Play procedure – to observe them. For this task, the parents role-play their first encounter with their future baby, represented by a doll. Two cases from a study on pregnancy after assisted reproductive technology are provided to illustrate how these behaviors manifest themselves. The observations from the first case suggest that expectant parents can offer the baby a coparental framework, whereas the observations from the second case show that opportunities for episodes of prenatal intuitive coparenting can be missed due to certain relationship dynamics. These kinds of observations deepen our knowledge of the prenatal emergence of the coparenting relationship and allow us to hone our strategies for intervening during pregnancy with couples who experience coparenting difficulties. Furthermore, these observations provide a novel and complementary perspective on prenatal intuitive parenting and coparenting behaviors.

## Introduction

Coparenting relates to the various ways parents coordinate with each other in childrearing (McHale and Lindahl, 2011). To date, coparenting research has focused on how parents share leadership, work together to resolve disagreements, and support or undermine one another in their joint role as architects of the family (McHale, 1995). A large body of evidence indicates that coparenting representations and behaviors influence child and family functioning (Teubert and Pinquart, 2010). Most research has assessed coparenting representations and behaviors after the child’s birth. Many studies have also explored coparenting during pregnancy by focusing on the representations the partners have of their future coparenting relationship, but did not examine the partners’ behaviors. These studies have demonstrated, among other things, that prenatal representations about the future family predict coparenting dynamics after birth (McHale et al., 2004; McHale and Rotman, 2007). Only a few studies have assessed prenatal coparenting behaviors. They have found that higher quality prenatal behavior is linked to more frequent supportive behavior, less frequent undermining behavior (Altenburger et al., 2014), and a more positive family alliance after birth (Carneiro et al., 2006). Coparenting is therefore believed to develop long before a child’s birth.

In this paper, we explore the intuitive nature of coparenting behaviors at the prenatal stage in greater depth. We posit that future parents *intuitively succeed in coordinating* their intuitive behaviors, which has not yet been studied: just as there are intuitive parenting behaviors (Papoušek and Papoušek, 1987), so too are there intuitive coparenting behaviors. Prenatal intuitive coparenting behaviors, as we propose to refer to them, consist of the subtle coordination between future parents when addressing their child. We use two cases to illustrate how future parents may exhibit these behaviors during a validated observational situation – the Prenatal Lausanne Trilogue Play procedure (Carneiro et al., 2006).

The aim of focusing on prenatal intuitive coparenting behaviors, from both a research and a clinical perspective, is to explore their nature. Learning more about them may also lead to the creation of preventive interventions for at-risk couples in the early stages of their transition to parenthood. Such interventions would address prenatal coparenting behaviors in order to work on the partners’ behavioral coordination and adjustment to their roles as parents.

### Research on Intuitive Parenting Behaviors

Intuitive parenting behaviors were first conceptualized and documented by Papoušek and Papoušek (1984, 1987). Using video-microanalytic research, they found that parents possess remarkable implicit communicative abilities (Papoušek, 2007) that allow them to adjust subtly to their infant’s capacities and promote its well-being and development (Papoušek et al., 2000). Intuitive parenting behaviors are considered preprogrammed and universal: they have been observed in different cultures, species, and genders, and in parents as well as non-parent adults (Bard, 1994; Schoppe-Sullivan et al., 2014). Children also display intuitive parenting behaviors, such as when a young child carefully holds a doll and uses baby talk. Recent research using neuroimaging data has shown that intuitive responses to baby signals are followed by processing in several regions of the brain (Maupin et al., 2015; Kringelbach et al., 2016).

Research on intuitive parenting behaviors has provided evidence that the context in which communication develops dynamically in infancy is intersubjective (Papoušek, 2007). During face-to-face interactions, parents express different behaviors, such as using a high-pitched and rhythmic voice, holding the baby at dialog distance, and looking and smiling at the baby, depending on the infant’s behavioral and affective states (Papoušek and Papoušek, 1987; Schoppe-Sullivan et al., 2014). The parents intuitively adjust their “multimodal communicative repertoire to the infant’s level of perceptual, integrative and communicative competence and know how to read and attribute meaning to their infant’s behavior” (Papoušek, 2007, p. 264). This intersubjective emotional communication is naturally also made possible by the infant’s perceptual and integrative capacities (Papoušek, 2007).

This knowledge of intuitive parenting behaviors and their effects on newborns was gained by experimentally eliminating the parental contingency using observational tasks such as the closed-eye paradigm (Papoušek and Papoušek, 1984). In this situation, the parent closes their eyes after a spontaneous face-to-face interaction of a few minutes. The results showed that 2–3 month-old babies reacted to this lack of reciprocity by withdrawing or protesting (Papoušek, 2007).

### Research on Prenatal Intuitive Parenting Behaviors

Originally, prenatal intuitive parenting behaviors were observed in the context of longitudinal studies documenting the development of the family alliance from the prenatal stage to toddlerhood (Lausanne Trilogue Play paradigm, LTP, Fivaz-Depeursinge and Corboz-Warnery, 1999; Fivaz-Depeursinge and Philipp, 2014). The prenatal coparenting alliance was assessed around the fifth month of pregnancy using the Prenatal Lausanne Trilogue Play procedure (Carneiro et al., 2006) in order to identify associations with the postnatal father, mother and baby alliance at different stages. The results showed that the higher the prenatal alliance, the higher the postnatal family alliance between the mother, father, and baby at 3 and 18 months after birth (Carneiro et al., 2006; Favez et al., 2006). Other studies have also used the Prenatal Lausanne Trilogue Play procedure. One showed that the mode of conception (assisted reproductive technology or spontaneous pregnancy) in a sample of 82 couples did not affect the quality of prenatal intuitive parenting (Darwiche et al., 2015). Another study, with a sample of 182 expectant parents, found less frequent intuitive parenting behaviors in fathers compared to mothers, but a moderate positive association between fathers’ and mothers’ intuitive parenting behaviors (Schoppe-Sullivan et al., 2014). That study also found that greater intuitive parenting behaviors in fathers (frequency, variety and intensity), associated with lower intuitive parenting behaviors in mothers, predicted greater developmentally appropriate activities from fathers with their 3-month-old babies. The last study used a modified version of the Prenatal Lausanne Trilogue Play procedure with a sample of 18 expectant couples. The parents were videotaped while watching a short video clip of a 4D routine ultrasound of their future baby. The data showed that mothers and fathers smiled more at the baby than at each other, and that mothers smiled more at the baby than fathers did (Ammaniti et al., 2014). In addition, the fathers talked more to their partner than to the baby. These differences may inform the different ways mothers and fathers become parents. The researchers also observed that some parents imitated the fetus’s movements while watching the video clip. For example, they imitated the hand, mouth, arm, and tongue movements, which the authors – Ammaniti et al. (2014) – interpreted as favoring the affiliation process between the parents and child.

The results of these studies on prenatal intuitive parenting document how parents activate specific behaviors during these observational procedures that may be precursors of their earliest communicational competencies with their future child.

### Exploration of Prenatal Intuitive Coparenting Behaviors

Prenatal intuitive parenting behaviors have been examined in several studies by observing and coding fathers’ and mothers’ individual behaviors while they either interacted with a doll representing their future baby or watched the fetal image of their baby through 4D ultrasound.

The next step is to explore whether prenatal intuitive *coparenting* behaviors can also be observed. We propose to define them as the intuitive behaviors of the parents-to-be toward the future baby, in coordination with each other. In other words, to paraphrase Mechthild Papoušek, prenatal intuitive coparenting is the capacity of the parents-to-be to “intuitively slow down, simplify, exaggerate, repeat, and vary facial expression and other behaviors in order to attune their behaviors” (Papoušek, 2007, p. 259), *while coordinating with each other*.

## Methods

To illustrate our exploratory work on prenatal intuitive coparenting behaviors, we will present two cases drawn from a longitudinal study on the transition from infertility to parenthood that aimed at describing the specific experience of pregnancy after assisted reproductive technology (Cairo et al., 2012; Darwiche et al., 2013). The cases come from the subsample of 33 couples that achieved pregnancy during the year following their first *in vitro* fertilization treatment. The women were primiparous, both partners were in their early thirties, 75% of them were married, and the average duration of infertility was 3 years. The study protocol received approval from the Ethical Committee of the Faculty of Biology and Medicine of the hospital.

**FIGURE 1:**
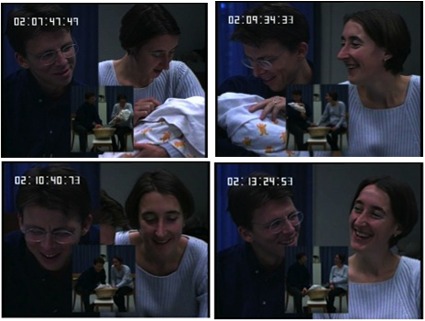
**The four parts of the Prenatal Lausanne Trilogue Play procedure.** (Part 1) Mother plays with the “baby.” (Part 2) Father plays with the “baby.” (Part 3) Parents play with the “baby” together. (Part 4) Parents talk together while “baby” is sleeping.

Multimodal measures were used (self-reported questionnaires, semi-structured interviews, and observational situations) to assess the partners’ emotional state, their marital quality, their acceptance of the infertility diagnosis, and the development of the family alliance. The partners did the Prenatal Lausanne Trilogue Play procedure between the 25th and 28th week of pregnancy, after the routine morphological ultrasound. Preceding the role-play was a warm-up phase consisting of a semi-structured interview about the expectant couple’s representations of their family-to-be (Carneiro et al., 2006). In the Prenatal Lausanne Trilogue Play procedure, the expectant parents are seated in a triangular configuration, with a basket as the third corner in the triangle. A facilitator asks the parents to role-play their first encounter with their baby, represented by a doll with no face that weighs as much as a real baby (referred to below as the “baby”). The instructions explain the four parts of the role-play (Cf. **Figure 1**): first, one of the parents plays with the “baby,” then the other, then the parents play with the “baby” together, and finally, they let the “baby” go to sleep and talk together about the experience they just had (Carneiro et al., 2006).

### Coding of Intuitive Coparenting Behaviors

In the above study, prenatal intuitive behaviors were independently coded for mothers and fathers as one component of the prenatal alliance (Cairo et al., 2012). In our exploratory work, prenatal intuitive *coparenting* behaviors were coded as follows:

(1) coparenting episodes were detected and delimited: the video was inspected completely at normal speed to get a first impression of the intuitive coparenting behaviors and to detect the episodes; then the videotape was viewed in slow motion to delimit each episode, defined as the smallest time period where intuitive coparenting behaviors were observable. For example, a mother is holding the “baby” and then the father moves toward them and holds the “baby” too. Then the parents rock the “baby” together for a while and the father moves back again. In this example, the episode starts when the father starts moving toward his partner and stops when he starts moving back.

For an episode to be considered an intuitive coparenting episode, both parents need to address and be involved with the “baby.” If they talk together about the “baby” or smile at each other, it may be an episode of coparenting but not an episode of *intuitive* coparenting. Finally, the frequency of episodes was calculated relative to the total duration of the role-play;

(2) the episodes were described and coded: each episode was described through a narrative that recounted the sequence of behaviors and detailed how and when the parents coordinated those behaviors. The narrative provides information on: (a) the type of behaviors simultaneously expressed by each parent (e.g., the mother is using baby-talk to communicate with the “baby” while the father is stroking the “baby’s” head), and the time they occurred; (b) an overall description of the parents’ gaze, facial expression and the direction their bodies are facing (e.g., parents look at each other, smile at each other, and are facing each other); (c) an evaluation of the emotional valence (i.e., attractiveness or averseness) of the episodes, such as whether they are warm and affectionate, instrumental (i.e., coordination of behaviors but no associated emotion), or tense and/or manifest intrusiveness (Del Vecchio and Sierro, 2015, Unpublished); (d) the duration of the episode.

## Case Illustrations

The first case illustrates episodes of warm and affectionate intuitive coparenting behaviors. The second case illustrates how opportunities for episodes of intuitive coparenting behaviors may be missed, such as when the parents fail to coordinate their behaviors to intuitively coparent a “baby.”

### Family A: Episodes of Warm and Affectionate Intuitive Coparenting Behaviors

The father starts the play: he takes the “baby” into his arms, rocks her gently and talks to her softly. After a while, he prepares to transition to the second part, in which the mother plays with the “baby.” During the transition, while the father is still holding the “baby,” he says, “Well, ok, you’re hungry… do you want to go eat?” Then he delicately moves the “baby” toward his wife, who takes the “baby” in her arms. Welcoming her with a large smile, she says to the “baby,”, “Hi, baby… do you recognize me? … I’m your mum.” At the same time the father adjusts the blanket, and then says, “Careful, she’s cold.” At that moment, the parents are coordinating their behaviors toward one another to get involved with the “baby” (Episode 1). Then father backs away and lets his wife interact with the “baby.”

Later in the role-play, the mother is playing with the “baby” and the father is looking on, smiling. At one point, the father approaches and asks the “baby,” “Just what is your name?” At that precise moment (Episode 2), both parents address the “baby”: the mother laughs and says, “My name is marmot,” while the father reaches out, touches the “baby,” and continues the conversation, saying, “They’re hidden. Her legs, her fingers…” After that, he stays involved but does not talk to or touch the “baby” anymore, so the episode of intuitive coparenting was considered over.

### Family B: A Missed Opportunity for Intuitive Coparenting Behaviors

The father starts the play. He holds the “baby” facing the mother and smiles and laughs embarrassedly. He quickly brushes away a tear from the corner of his eye – overcome by the situation. The mother fails to notice and they both laugh awkwardly and are uncomfortable with the task. The father continues holding the “baby” and adjusting the small blanket covering it, all the while looking at the “baby.” After a time, he hands the “baby” carefully to the mother. She faces the “baby” toward her, with her back to the father. She notices the father watching her and says to him in a low voice, “You shouldn’t look at me.” The father fidgets a lot and scratches. Then the mother says to the “baby,” “Hi, you,” and again says to her husband, “Don’t look at me.” The husband says, “No, I’m not looking at you,” turns away and looks up (he doesn’t really know where to look), and then looks straight ahead. It is unclear in this episode whether the father would have wanted to talk to the “baby” together with the mother for an episode of intuitive coparenting, but we observe how the mother inhibits any desire the father might have had to coordinate by telling him twice not to look at her.

## Discussion and Conclusion

These two contrasting cases illustrate the kinds of observations we can make when we uncover a subtle multimodal coordination between the parents. They coordinate their gestures and their actions to get involved with the “baby”; their movements follow a common rhythm, and their speech is complementary so that they can address the “baby” together. They appear to be intuitively offering a coparental framework to the “baby.” In contrast, the second family reminds us that intuitive coparenting cannot be taken for granted in every situation. The mother does not let the father come close and does not give him the opportunity to join her in interacting with the “baby.” It is possible that the mother wanted to apply the role-play directions very (too) strictly. The instructions were to take turns interacting with the “baby” on their own. It is also possible that there were tensions in the couple that spilled over into the role-play.

This exploratory work on intuitive coparenting raises several questions. The first of these is what we can learn about the development of communication between parents and babies from exploring intuitive coparenting compared with observing intuitive parenting. Intuitive coparenting requires adults to coordinate to take care of a newborn. This coordination could be considered a cooperative behavior that is mutually beneficial to the parents and the baby (West et al., 2011) for the development of their communication and, more broadly, their experience of being together as a family. Future work should go beyond these observations in order to deepen our understanding of how both emergent and planned coordination between the partners makes joint action possible in this prenatal task (Knoblich et al., 2011). The observed coordination could then be compared with the future parents’ feelings of connectedness. Some studies have shown that greater “teamness” and harmoniousness (Marsh et al., 2009) and increased affiliation and liking the partner more (Miles et al., 2009) are associated with more coordination, expressed by the individuals interacting with one another (Knoblich et al., 2011).

Our observations may also reinforce the idea that coparenting emerges early – at the prenatal stage. The question of whether these behaviors should be considered innate or learned is not the topic of this paper, but given that intuitive parenting is considered preprogrammed (Papoušek et al., 1992), we can hypothesize about whether prenatal intuitive coparenting behaviors might also be preprogrammed.

Another question raised by this exploratory work is about what differences there might be between the intuitive parenting behaviors observed in a dyadic (i.e., parent–child) situation and a triadic situation where both parents interact with the baby. From the baby’s perspective the subjective experience is drastically different, because the parents can behave differently and the triadic situation can amplify or inhibit their individual intuitive parenting behaviors. In addition, the triadic situation gives the baby the opportunity to experience the parents coordinating to get involved with him or her. Such an experience can help the baby develop his or her triangular competence in engaging in interactions with two individuals at the same time, and in detecting the contingent reactions between him or herself and the two parents (Fivaz-Depeursinge and Corboz-Warnery, 1999). Finally, from a clinical perspective, it could be important to provide support to the parents experiencing difficulty with prenatal intuitive coparenting. That support could act as a protective and developmentally vital force for future coparenting.

## Author Contributions

JD: substantial contribution to the conception of the work and the interpretation of the results; drafted the work. EF-D: substantial contribution to the conception of the work and the interpretation of the results; substantial revision of the work. AC-W: substantial contribution to the conception of the work and the interpretation of the results; substantial revision of the work. The three authors agreed to be accountable for all aspects of the work in ensuring that questions related to the accuracy or integrity of any part of the work are appropriately investigated and resolved.

## Conflict of Interest Statement

The authors declare that the research was conducted in the absence of any commercial or financial relationships that could be construed as a potential conflict of interest.
